# Postoperative vascular compromise after digital replantation or revascularization: timing, risk factors, and salvage outcomes in a single-center retrospective cohort

**DOI:** 10.3389/fmed.2026.1926596

**Published:** 2026-08-26

**Authors:** Yao Qian, Keyue Yang, Xinkun He, Xiaofeng Teng, Zhen Xu, Jie Zhang

**Affiliations:** 1 Department of Hand Microsurgery and Plastic Reconstructive Surgery, Ningbo No. 6 Hospital, Ningbo City, Zhejiang Province, China; 2 Ningbo Clinical Research Center for Orthopedics, Sports Medicine & Rehabilitation, Ningbo, Zhejiang, China

**Keywords:** digital replantation, digital revascularization, nursing surveillance, retrospective cohort, salvage, vascular compromise, venous congestion

## Abstract

**Background:**

Postoperative vascular compromise remains a clinically important threat after digital replantation or revascularization, but its timing, clinical presentation, risk factors, and post-compromise salvage profile are often less systematically reported than final survival.

**Methods:**

We performed a single-center retrospective cohort study using digit-level records. The primary outcome was postoperative vascular compromise at the digit level. Compromise type, the operation-start–referenced interval to first documentation of compromise, recorded management components, and salvage success, defined as complete survival or partial survival with digit preservation and without secondary amputation, were summarized. Logistic regression with patient-cluster robust standard errors was used for the vascular-compromise model; salvage models were exploratory complete-case analyses.

**Results:**

This single-center retrospective study enrolled 671 digits from 484 patients. Postoperative vascular compromise occurred in 128 of 671 digits (19.1%). Venous congestion was the most common presentation (71.9%), followed by arterial insufficiency (21.1%) and mixed compromise (7.0%). The median operation-start–referenced interval to first documentation of vascular compromise was 15.88 h (IQR, 8.03–34.52), and 112 of 128 events (87.5%) were first documented within 48 h after the recorded operation-start timestamp. In the parsimonious multivariable vascular-compromise model, complete amputation (OR, 2.853; 95% CI, 1.780–4.575), vein grafting (OR, 2.963; 95% CI, 1.615–5.437), and longer operative duration (OR per hour, 1.079; 95% CI, 1.010–1.152) were associated with higher odds of compromise, whereas a greater number of arterial anastomoses was associated with lower odds (OR, 0.526; 95% CI, 0.331–0.835). Among compromise digits, salvage success was achieved in 81 of 128 digits (63.3%). Any recorded thrombolysis was associated with lower odds of salvage success in an exploratory complete-case model (*N* = 84; OR, 0.243; 95% CI, 0.089–0.662), a finding likely affected by confounding by indication.

**Conclusions:**

In this single-center retrospective cohort, approximately one in five replanted or revascularized digits developed postoperative vascular compromise, most commonly venous congestion. Complete amputation, vein grafting, longer operative duration, and fewer arterial anastomoses were associated with compromise and may help identify digits requiring intensified early surveillance. Post-compromise treatment-outcome findings should be interpreted as exploratory associations rather than causal effects.

## 1 Introduction

Traumatic digital amputation is a severe hand injury that can permanently impair digit length, sensibility, appearance, fine motor function, and return to daily activities. Digital replantation aims to restore perfusion and preserve the amputated part when patient factors, the digit involved, injury mechanism, ischemia, and expected rehabilitation burden make replantation appropriate (1). Outcomes after replantation extend beyond survival and include sensibility, appearance, function, and return to daily activities (2). Even when arterial inflow is restored in the operating room, the early postoperative period remains vulnerable because small-caliber vessels, intimal injury, edema, vasospasm, and venous outflow limitations can compromise the replant.

Published survival rates after digit replantation vary widely across injury types, centers, and levels of amputation, as summarized in a recent narrative review (3). Earlier meta-analytic evidence also showed substantial heterogeneity in digit survival after replantation (4). Distal digital amputations represent a distinct subgroup with different survival and functional considerations (5). Predictors of survival reported in previous studies include injury mechanism, ischemia, amputation level, patient factors, and operative factors (6). A separate review of predictive factors similarly emphasized that survival after replantation depends on multiple patient-, injury-, and treatment-level variables (7). Contemporary cohort and database studies further suggest that case selection and injury characteristics influence replantation attempt and success rates (8). National database evidence from Japan also supports the importance of patient and injury features when interpreting replantation outcomes (9). The number and balance of repaired arteries and veins may also influence replant survival (10). These data have shaped preoperative decision-making, but they are less informative for ward-level surveillance after the digit has already been replanted. For clinicians and nurses who monitor color, temperature, capillary refill, pain, bleeding, and swelling hour by hour, the practical question is not only whether a digit survives, but when vascular compromise tends to appear and which digits are most likely to require attention. Postoperative management reviews similarly emphasize close monitoring of perfusion and timely rehabilitation after replantation or revascularization (11).

Postoperative vascular compromise is an early postoperative perfusion event that is clinically distinct from final necrosis or secondary amputation, although unresolved compromise may contribute to these downstream outcomes. A recent complications review similarly frames vascular compromise as a major postoperative complication requiring prompt recognition and management (12). It marks a time-sensitive deterioration in perfusion and usually triggers escalation of warming, positioning, anticoagulation adjustment, antispasm therapy, bleeding or leech strategies, thrombolysis, or re-exploration. Evidence for perioperative antithrombotic strategies remains heterogeneous and is often based on low-level comparative data (13). Postoperative digit and hand replantation protocols also vary across institutions, underscoring the need for clinically practical surveillance data (14). A Nordic survey likewise showed substantial variation in postoperative monitoring, anticoagulation, dressings, bed rest, therapy, and follow-up after hand replantation (15).

A second gap concerns phenotype. Venous congestion, arterial insufficiency, and mixed compromise differ in bedside appearance, plausible mechanism, and response to treatment. Yet many datasets collapse these events into survival or failure endpoints. This limits the ability to design practical surveillance pathways. A digit that fails because of early venous outflow obstruction may require different monitoring and response thresholds than a digit that develops delayed arterial insufficiency.

A third gap concerns denominators. Replantation studies often mix patient-level baseline characteristics with digit-level surgical exposures, which can lead to unstable interpretation when one patient contributes more than one digit. A digit-level analysis should account for clustering within patients and should distinguish the population used for the compromise-occurrence model from the smaller compromise subset used for post-compromise salvage analysis.

The present study addresses these practical gaps using a single-center retrospective cohort of digital replantation and revascularization. The primary objective was to describe the incidence of postoperative vascular compromise and identify digit-level and operative factors associated with compromise occurrence. Secondary objectives were to characterize compromise phenotype and operation-start-referenced timing, summarize recorded management components, and explore factors associated with post-compromise salvage success among digits that developed compromise. Because the study is observational and treatment allocation was not randomized, all treatment-outcome analyses were considered exploratory and hypothesis-generating.

## 2 Methods

### 2.1 Study design and participants

This was a single-center retrospective cohort study conducted in the Department of Hand Surgery at Ningbo No. 6 Hospital, using one digit-level analytic record per replanted or revascularized digit. The study was reported in accordance with STROBE reporting principles for observational studies. Consecutive patients who underwent digital replantation or revascularization between January 2024 and December 2025 were screened for eligibility. The study protocol was reviewed and approved by the Ethics Committee of Ningbo No. 6 Hospital before the study period (Approval 2023-24[K]). Data were abstracted from existing electronic medical records, operative records, nursing surveillance records, treatment records, and follow-up records.

Consecutive patients who underwent microsurgical digital replantation or revascularization for traumatic complete digital amputation or incomplete amputation/devascularization at our center during the study period were eligible. The primary analytic unit was the digit: 671 digits were contributed by 484 patients, and 110 patients contributed two or more digits. Each digit was assigned a unique digit-level identifier. Patient-level baseline characteristics were summarized once per patient, whereas digit-level surgical variables and vascular-compromise outcomes were analyzed per digit. Eligible digits were required to have postoperative clinical records sufficient to ascertain vascular-compromise status, including medical records, nursing surveillance records, operative notes, re-exploration records, or treatment records.

Digits were excluded if they were treated for non-traumatic amputation, elective amputation, tumor, infection, congenital deformity, or chronic ischemia; if no replantation or revascularization was attempted; if only revision amputation, stump closure, local flap coverage, or non-microsurgical reconstruction was performed; if the case involved major hand, wrist, forearm, or upper-limb replantation without separable digit-level outcomes; if a unique patient or digit identifier could not be constructed; or if postoperative vascular-compromise status could not be determined after chart review. Digits with intraoperative failure to restore initial perfusion were not included in the postoperative vascular-compromise risk set and were summarized separately when identifiable.

For construction of the final cohort, records lacking sufficient baseline or outcome information to determine eligibility or vascular-compromise status were excluded. For subsequent table- or model-specific analyses, records were excluded only from the corresponding analysis when the required predictor or treatment variable was missing or internally inconsistent. Complete-case denominators are reported in the relevant tables, table footnotes, and Results section.

### 2.2 Surgical procedure and postoperative management

Microsurgical replantation or revascularization included debridement, skeletal fixation or shortening when needed, arterial repair, venous repair when feasible, vein grafting when direct tension-free repair was not possible, and tendon or nerve repair when clinically feasible. No uniform written protocol or protocol-mandated escalation algorithm for postoperative vascular compromise was used during the study period. Postoperative bedside surveillance was performed by the clinical care team and was based on recorded assessments of digit color, temperature, turgor or swelling, capillary refill, pain, pin-prick or wound bleeding characteristics, and Doppler signal when documented. Warming or positioning, anticoagulation adjustment, antispasm therapy, bleeding or leech-based decompression, thrombolysis, and operative re-exploration were individualized according to compromise phenotype, severity, persistence, suspected mechanism, and surgeon judgment. Detailed information on drug names, doses, administration routes, treatment duration, anticoagulation targets, bleeding or leech schedules, and prespecified criteria or timing for operative re-exploration was not consistently documented in a structured form and could not be reliably reconstructed. Consequently, treatment was analyzed only as the presence or absence of recorded, non-mutually exclusive management components.

### 2.3 Outcomes and variables

The primary outcome was postoperative vascular compromise at the digit level, defined as documented postoperative circulatory compromise of a replanted or revascularized digit after initial perfusion had been restored. Venous congestion was classified mainly by dusky or purple color, swelling or increased turgor, abnormal capillary refill, dark or brisk bleeding on pin-prick or wound assessment, and preserved or relatively warm temperature when recorded. Arterial insufficiency was classified mainly by pallor or gray discoloration, reduced temperature, delayed or absent capillary refill, weak or absent bleeding, and reduced Doppler signal when documented. Mixed compromise was recorded when arterial insufficiency and venous congestion features coexisted in the same episode. The first documented vascular-compromise episode per digit after initial perfusion had been restored was used for the operation-start-referenced timing analysis; recurrent events in the same digit were not counted as separate compromise digits.

The timing variable was defined as the elapsed time, in hours, from the recorded operation-start timestamp to the first documented abnormal-perfusion or vascular-compromise record after initial perfusion had been restored. The operation-start timestamp was the only consistently available time origin in the retrospective records. Because uniform timestamps for reperfusion, completion of the final vascular anastomosis, completion of surgery, or initiation of postoperative monitoring were unavailable, this interval included a variable intraoperative period and was not interpreted as standardized postoperative elapsed time or biological onset time. For two records with missing or evidently erroneous electronic interval values, the interval was recalculated from the recorded operation-start timestamp to the first available abnormal-perfusion or clinician-confirmed compromise timestamp. The interval was summarized continuously and in mutually exclusive categories of 0 to <6, 6 to <12, 12 to <24, 24 to ≤48, and >48 h.

The main post-compromise outcome was derived salvage success. Final digit-survival status was determined from the last available discharge or follow-up record in the electronic medical record; a uniform fixed follow-up time point was not available because of the retrospective design. Derived salvage success was defined as complete survival or partial survival with digit preservation and without documented secondary amputation. Derived salvage failure was defined as complete necrosis, documented loss or non-salvage of the digit, secondary amputation, or raw salvage-failure documentation when structured survival-status fields were insufficient. When source outcome information conflicted, secondary amputation or documented loss of the digit overrode partial-survival coding.

Candidate predictors included age, sex, smoking status, diabetes, hypertension, injury mechanism, contamination grade, total ischemia time, amputation type, Tamai zone, actual number of completed arterial and venous anastomoses per digit, vein grafting, arterial grafting, and operative duration. Operative duration and total ischemia time were recorded at the operation or patient level and were assigned to each digit treated during that operation. In multi-digit procedures, these values may therefore represent overall case complexity rather than independent digit-specific exposures. To evaluate whether duplication of operation-level exposure values influenced the operative-duration estimate, we performed a sensitivity analysis restricted to operations involving a single treated digit.

### 2.4 Statistical analysis

Continuous variables were summarized as median with interquartile range, and categorical variables as counts and percentages. For univariable comparisons between compromise and no-compromise groups, continuous variables were compared using the Mann–Whitney *U* test or Wilcoxon rank-sum test as appropriate, and categorical variables were compared using the chi-square test or Fisher exact test according to expected cell counts. Candidate variables were selected according to clinical relevance, prior evidence, data availability, descriptive univariable findings, and events-per-parameter considerations rather than automatic stepwise selection. The revised primary multivariable model was restricted to five prespecified predictors: complete amputation, severe contamination, number of arterial anastomoses, vein grafting, and operative duration. Patient-level and digit-level denominators were kept separate, and logistic regression models used patient-cluster robust standard errors to account for multiple digits within the same patient. The single-digit sensitivity analysis used one record per operation and refitted the prespecified vascular-compromise model. Severe contamination was modeled against all other recorded or unknown contamination grades in the primary model; a sensitivity analysis excluding unknown contamination status was also reviewed. Missing data were handled by complete-case analysis within each model; no imputation was performed. Any thrombolysis was defined as thrombolysis recorded at any time during post-compromise treatment, whereas thrombolysis as an initial management component was reported separately according to the documented initial post-compromise management in the electronic records. Exploratory logistic models within the compromise cohort estimated associations of the operation-start-referenced interval to first documentation of compromise, compromise presentation, and thrombolysis treatment with salvage success and were not intended as validated prediction models. All treatment-outcome results were interpreted as associations, not causal effects, because treatment decisions were clinically determined. All analyses were performed using R software, version 4.5.3 (R Foundation for Statistical Computing, Vienna, Austria). A two-sided *p* value <0.05 was considered statistically significant.

For model diagnostics, the functional form of operative duration was assessed by comparing the linear specification with a restricted cubic spline using three knots at the 10th, 50th, and 90th percentiles. Because the number of completed arterial anastomoses had only two observed values in the final model dataset, its per-anastomosis specification was equivalent to a categorical comparison of two vs. one completed arterial anastomosis. Multicollinearity was assessed using variance inflation factors, with VIF > 5 considered potentially concerning.

## 3 Results

### 3.1 Study population and analytic denominators

**Figure 1:**
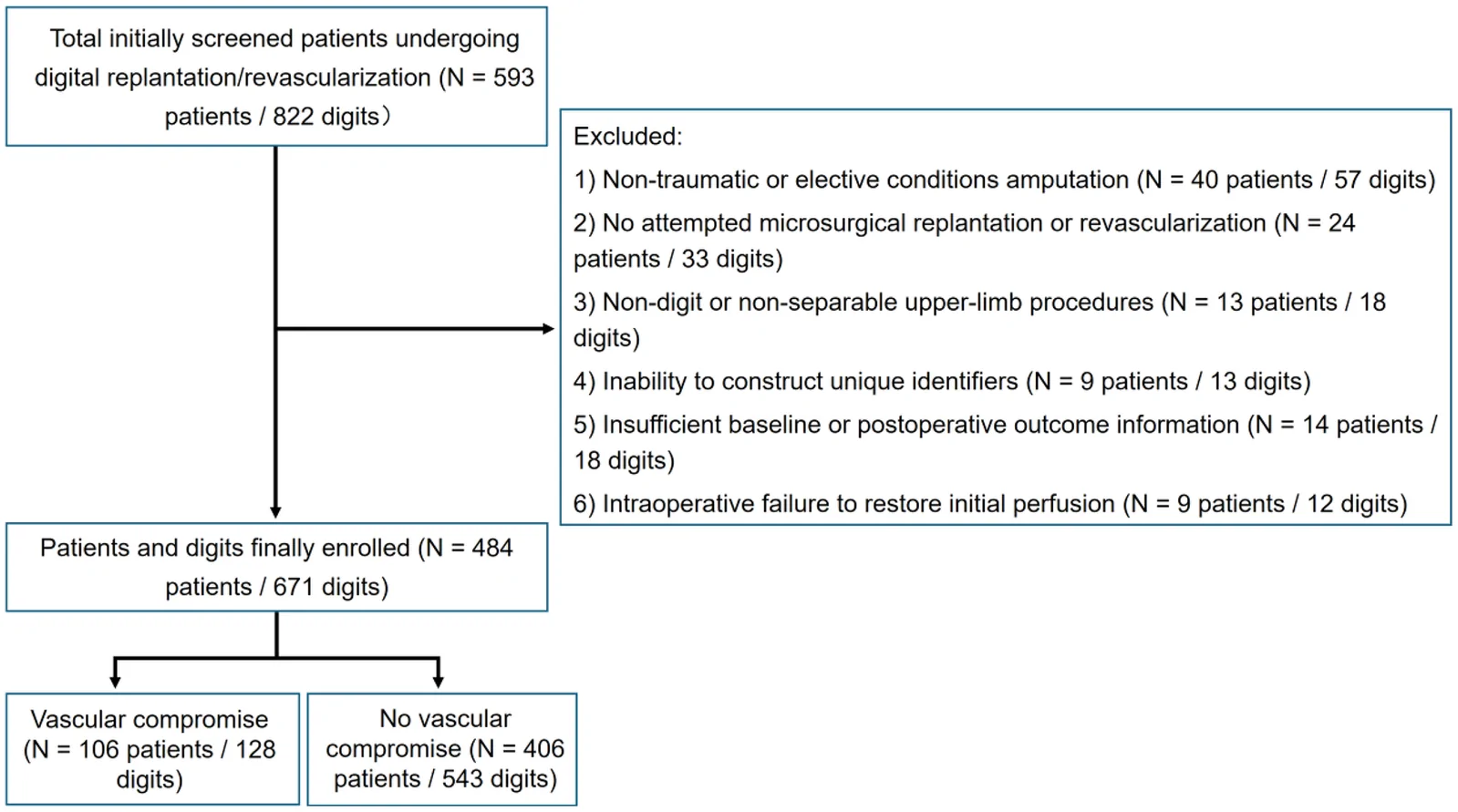
Flow chart of this study.

A total of 593 patients, representing 822 potentially eligible digits, were initially screened (Figure 1). Patients and digits were excluded sequentially for non-traumatic or elective conditions (40 patients/57 digits), absence of attempted microsurgical replantation or revascularization (24 patients/33 digits), non-digit or non-separable upper-limb procedures (13 patients/18 digits), inability to construct unique identifiers (9 patients/13 digits), insufficient baseline or postoperative outcome information (14 patients/18 digits), and intraoperative failure to restore initial perfusion (9 patients/12 digits). The final postoperative risk set comprised 484 patients and 671 digits. Among these, 128 digits developed postoperative vascular compromise and were contributed by 106 patients, whereas 543 digits had no documented postoperative vascular compromise and were contributed by 406 patients. Because some patients contributed multiple digits, these patient counts were not mutually exclusive: 28 patients contributed both compromise and no-compromise digits.

### 3.2 Patient-level baseline characteristics

At the patient level, median age was 49 years (IQR, 36–56), and 389 of 484 patients (80.4%) were male (Table 1). Median body mass index was 23.44 kg/m^2^ (IQR, 21.37–26.29). Former or current smoking was recorded in 33 patients (6.8%), diabetes in 24 (5.0%), and hypertension in 61 (12.6%). Most patients contributed one replanted digit (374/484, 77.3%), whereas 110 patients (22.7%) contributed two or more digits.

**Table 1:** Patient-level baseline characteristics (*N* = 484 patients).

Characteristic	Overall (*N* = 484 patients)
Age (years), median [IQR]	49 [36, 56]
BMI (kg/m^2^), median [IQR]	23.44 [21.37, 26.29]
Sex, *n* (%)	
Male	389 (80.4%)
Female	95 (19.6%)
Smoking status, *n* (%)	
Former/current smoking	33 (6.8%)
Never	451 (93.2%)
Diabetes, *n* (%)	24 (5.0%)
Hypertension, *n* (%)	61 (12.6%)
Number of replanted/revascularized digits per patient	
One digit per patient	374 (77.3%)
≥2 digits per patient	110 (22.7%)
Operative duration (h), median [IQR]	2.25 [1.33, 3.83]
Total ischemia time (h), median [IQR]	4.83 [3.83, 6.25]

This table is summarized at the patient level. Operative duration and total ischemia time are patient- or operation-level summaries.

### 3.3 Digit-level characteristics by vascular-compromise status

Postoperative vascular compromise occurred in 128 of 671 digits (19.1%). In the digit-level comparison (Table 2), laterality and digit type were similarly distributed between the no-compromise and compromise groups (*p* = 0.681 and *p* = 0.705, respectively). Injury mechanism did not differ significantly between groups (*p* = 0.299), and contamination grade showed no statistically significant difference (*p* = 0.082). Complete amputation was more frequent among compromise digits than no-compromise digits (46.9% vs. 21.2%; *p* < 0.001). Vascular compromise occurred in 60 of 175 complete amputations (34.3%) and 68 of 496 incomplete amputation or revascularization digits (13.7%). Tamai zone distribution did not differ significantly (*p* = 0.094). The actual number of completed arterial anastomoses per digit was lower in the compromise group (1.00 [IQR, 1.00–1.00]) than in the no-compromise group (1.00 [IQR, 1.00–2.00]; *p* = 0.008), whereas venous anastomosis count was not significantly different (*p* = 0.251). Vein grafting was more frequent among compromise digits (26/128, 20.3% vs. 32/543, 5.9%; *p* < 0.001), whereas arterial grafting and tendon repair did not differ significantly between groups (*p* = 0.285 and *p* = 0.090, respectively).

**Table 2:** Digit-level characteristics by postoperative vascular-compromise status (*N* = 671 digits).

Variable	Overall (*N* = 671)	No compromise (*N* = 543)	Compromise (*N* = 128)	*P* value
Laterality, *n* (%)				0.681
Left	336 (50.1)	274 (50.5)	62 (48.4)	
Right	335 (49.9)	269 (49.5)	66 (51.6)	
Digit type, *n* (%)				0.705
Thumb	113 (16.8)	90 (16.6)	23 (18.0)	
Index finger	178 (26.5)	145 (26.7)	33 (25.8)	
Middle finger	171 (25.5)	144 (26.5)	27 (21.1)	
Ring finger	135 (20.1)	106 (19.5)	29 (22.7)	
Little finger	74 (11.0)	58 (10.7)	16 (12.5)	
Mechanism, *n* (%)				0.299
Sharp cut	81 (12.1)	71 (13.1)	10 (7.8)	
Crush injury	146 (21.8)	119 (21.9)	27 (21.1)	
Avulsion	26 (3.9)	18 (3.3)	8 (6.2)	
Machine/complex injury	384 (57.2)	308 (56.7)	76 (59.4)	
Other/unknown	34 (5.1)	27 (5.0)	7 (5.5)	
Contamination grade, *n* (%)				0.082
Moderate	148 (22.1)	111 (20.4)	37 (28.9)	
None/mild	10 (1.5)	9 (1.7)	1 (0.8)	
Severe	421 (62.7)	352 (64.8)	69 (53.9)	
Unknown	92 (13.7)	71 (13.1)	21 (16.4)	
Amputation, *n* (%)				<0.001
Incomplete amputation	496 (73.9)	428 (78.8)	68 (53.1)	
Complete amputation	175 (26.1)	115 (21.2)	60 (46.9)	
Tamai zone, *n* (%)				0.094
Tamai 3	237 (35.3)	179 (33.0)	58 (45.3)	
Tamai 4	231 (34.4)	191 (35.2)	40 (31.2)	
Tamai 2	141 (21.0)	121 (22.3)	20 (15.6)	
Tamai 5	54 (8.0)	46 (8.5)	8 (6.2)	
Tamai 1	8 (1.2)	6 (1.1)	2 (1.6)	
Arterial anastomosis, median [IQR]	1.00 [1.00, 2.00]	1.00 [1.00, 2.00]	1.00 [1.00, 1.00]	0.008
Venous anastomosis, median [IQR]	2.00 [1.00, 2.00]	2.00 [1.00, 2.00]	1.00 [1.00, 2.00]	0.251
Vein graft, *n* (%)				<0.001
No	613 (91.4)	511 (94.1)	102 (79.7)	
Yes	58 (8.6)	32 (5.9)	26 (20.3)	
Arterial graft, *n* (%)				0.285
No	658 (98.1)	534 (98.3)	124 (96.9)	
Yes	13 (1.9)	9 (1.7)	4 (3.1)	
Tendon repair, *n* (%)				0.090
No	346 (51.6)	270 (49.7)	76 (59.4)	
Flexor tendon	67 (10.0)	59 (10.9)	8 (6.2)	
Extensor tendon	100 (14.9)	87 (16.0)	13 (10.2)	
Both	158 (23.5)	127 (23.4)	31 (24.2)	

This table is summarized at the digit level; patients with multiple digits may contribute multiple records. Arterial and venous anastomosis counts refer to actual completed anastomoses per digit, not attempted repairs.

In the complete-amputation subgroup, 60 of 175 digits developed postoperative vascular compromise (34.3%), including 19 arterial insufficiency, 36 venous congestion, and 5 mixed-compromise events; salvage success was achieved in 30 of these 60 compromise digits (50.0%). Among 496 incomplete amputation or devascularization digits undergoing revascularization, 68 developed compromise (13.7%), including 8 arterial insufficiency, 56 venous congestion, and 4 mixed-compromise events; salvage success was achieved in 51 of these 68 compromise digits (75.0%).

### 3.4 Multivariable model for vascular compromise

In the revised parsimonious multivariable patient-cluster robust model (Table 3; complete-case *N* = 671, events = 128), complete amputation (OR, 2.853; 95% CI, 1.780–4.575; *p* < 0.001), vein grafting (OR, 2.963; 95% CI, 1.615–5.437; *p* < 0.001), and longer operative duration (OR per hour, 1.079; 95% CI, 1.010–1.152; *p* = 0.025) were associated with higher odds of postoperative vascular compromise. A greater number of completed arterial anastomoses was associated with lower odds of compromise (OR, 0.526; 95% CI, 0.331–0.835; *p* = 0.006). Severe contamination showed an inverse association (OR, 0.536; 95% CI, 0.341–0.844; *p* = 0.007). In the single-digit sensitivity analysis, 380 digits/operations from 377 patient clusters were included, with 77 postoperative vascular-compromise events and 303 non-events. The association between operative duration and postoperative vascular compromise remained positive in direction but was no longer statistically significant (OR per hour, 1.112; 95% CI, 0.905–1.365; *p* = 0.313).

**Table 3:** Multivariable logistic model for postoperative vascular compromise.

Variable	OR	95% CI	*P* value	Complete-case N
Complete amputation	2.853	1.780–4.575	<0.001	671
Severe contamination	0.536	0.341–0.844	0.007	671
Arterial anastomoses	0.526	0.331–0.835	0.006	671
Vein graft	2.963	1.615–5.437	<0.001	671
Operative duration, h	1.079	1.010–1.152	0.025	671

The model included 671 complete-case digits, 128 events, 543 non-events, and five predictors excluding the intercept (25.6 events per predictor). Patient-cluster robust standard errors were used. The modeled event was postoperative vascular compromise; no postoperative vascular compromise was the outcome reference. Reference categories were incomplete amputation or revascularization for complete amputation, all other recorded or unknown contamination grades for severe contamination, and no vein graft for vein grafting. The arterial-anastomosis odds ratio represents two vs. one completed arterial anastomosis, and the operative-duration odds ratio represents a one-hour increase. Model diagnostics showed no evidence of operative-duration nonlinearity and no material multicollinearity.

Model diagnostics showed no evidence of departure from linearity for operative duration (likelihood-ratio chi-square = 1.219; *p* for nonlinearity = 0.270). The arterial-anastomosis variable had only two observed values, so the per-anastomosis specification was equivalent to comparing two vs. one completed arterial anastomosis. The maximum variance inflation factor was 1.154, indicating no material multicollinearity among the included predictors.

### 3.5 Compromise phenotype, operation-start-referenced timing, and recorded management

Among 128 compromise digits, venous congestion was most common (92/128, 71.9%), followed by arterial insufficiency (27/128, 21.1%) and mixed compromise (9/128, 7.0%) (Table 4). Operation-start-referenced timing data were available or derived for all 128 compromise digits. The median operation-start-referenced interval to first documentation of vascular compromise was 15.88 h (IQR, 8.03–34.52), mean time was 23.93 h (SD, 21.36), and the observed range was 0.87–98.53 h. A total of 112 of 128 events (87.5%) were first documented within 48 h after the recorded operation-start timestamp. Recorded non-mutually exclusive initial management components included conservative warming or positioning (115/128, 89.8%), anticoagulation adjustment (19/128, 14.8%), antispasm treatment (12/128, 9.4%), thrombolysis as an initial component (13/128, 10.2%), bleeding (4/128, 3.1%), leech treatment (0/128, 0.0%), and exploration as an initial component (3/128, 2.3%). Any thrombolysis recorded at any point during post-compromise treatment was present in 39 of 84 complete treatment records (46.4%). These treatment variables represented recorded management components rather than standardized protocols.

**Table 4:** Postoperative vascular-compromise phenotype, operation-start-referenced timing, and recorded management components.

Measure	Result
Postoperative vascular compromise, *n* (%)	128/671 (19.1)
Vascular compromise, *n* (%)	
Venous compromise	92/128 (71.9)
Arterial compromise	27/128 (21.1)
Mixed compromise	9/128 (7.0)
Operation-start-referenced interval to first documentation of compromise (h), median [IQR]	15.88 [8.03, 34.52]; mean 23.93 (SD 21.36); range 0.87–98.53
Operation-start-referenced interval category, *n* (%)	
0 to <6 h	21 (16.4); salvage success 15/21 (71.4)
6 to <12 h	26 (20.3); salvage success 20/26 (76.9)
12 to <24 h	34 (26.6); salvage success 19/34 (55.9)
24 to ≤48 h	31 (24.2); salvage success 16/31 (51.6)
>48 h	16 (12.5); salvage success 11/16 (68.8)
First management, *n* (%)	
Conservative warming/positioning	115/128 (89.8)
Anticoagulation adjustment	19/128 (14.8)
Antispasm therapy	12/128 (9.4)
Thrombolysis as initial management component	13/128 (10.2)
Bleeding as initial management component	4/128 (3.1)
Leech treatment as initial management component	0/128 (0.0)
Exploration as initial management component	3/128 (2.3)
Any thrombolysis recorded during post-compromise treatment	39/84 (46.4)

Timing was defined as the interval from the recorded operation-start timestamp to the first documented abnormal-perfusion or vascular-compromise record after initial perfusion had been restored. This interval included a variable intraoperative period and should not be interpreted as standardized postoperative elapsed time or biological onset time. For two records with missing or evidently erroneous electronic interval values, the interval was recalculated using the recorded operation-start timestamp and the first available abnormal-perfusion or clinician-confirmed compromise timestamp. Management components are non-mutually exclusive.

### 3.6 Post-compromise outcomes and exploratory salvage models

Table 5 summarizes clinical outcomes among the 128 compromise digits and reconciles the derived salvage outcome with the secondary-amputation field. Derived salvage success was achieved in 81 of 128 compromise digits (63.3%), whereas 47 digits (36.7%) met the derived salvage-failure definition. Among the 47 derived failures, 31 had a recorded digit-level secondary-amputation indicator, whereas the remaining 16 were further reconciled in Table 5 as structured final-status failures or raw salvage-failure fallback cases. Partial necrosis with digit preservation was recorded in 15 salvage-success digits. Table 6 compares selected factors by salvage status. Compromise type was not significantly associated with salvage status (*p* = 0.241), and the operation-start-referenced interval to first documentation of compromise did not differ significantly between failures and successes (median, 20.25 [IQR, 11.98–36.58] vs. 14.33 [IQR, 7.33–33.70] h; *p* = 0.187). Bleeding/leech-based treatment and re-exploration were also not significantly associated with salvage status (*p* = 1.000 and *p* = 0.558, respectively). Re-exploration status was available for 122 of 128 compromise digits, and 3 of 122 digits (2.5%) underwent operative re-exploration. Reasons for not performing re-exploration were not consistently documented and could not be reliably categorized. In the 84 complete treatment records shown in Table 6, any recorded thrombolysis was more frequent among failures than successes (21/33 vs. 18/51; *p* = 0.011).

**Table 5:** Outcomes among digits with postoperative vascular compromise (*N* = 128 compromise digits).

Outcome	Result
Derived salvage success	81/128 (63.3)
Derived salvage failure	47/128 (36.7)
Failures with recorded digit-level secondary-amputation indicator	31/47 (66.0)
Other derived failures without separate digit-level secondary-amputation indicator	16/47 (34.0)
Structured final-status failure without separate secondary-amputation indicator	8/16 (50.0)
Raw salvage-failure fallback without reliable further digit-level subdivision	8/16 (50.0)
Partial necrosis with digit preservation among salvage-success digits	15/81 (18.5)
Infection among compromise digits	1/128 (0.8)
Re-exploration with recorded denominator	3/122 (2.5)

Derived salvage success was defined as complete survival or partial survival with digit preservation and without documented secondary amputation. Derived salvage failure included complete necrosis, documented loss or non-salvage of the digit, recorded secondary amputation, or raw salvage-failure documentation when structured survival-status fields were insufficient. Secondary amputation refers to the structured digit-level secondary-amputation indicator; therefore, derived salvage failure can exceed the number of records with this specific indicator. Final digit-survival status was determined from the last available discharge or follow-up record in the electronic medical record; no uniform fixed follow-up time point was available. Among the 16 derived salvage failures without a separate digit-level secondary-amputation indicator, 8 were classified from structured final digit-survival status and 8 from the raw salvage-failure fallback because the available structured survival-status information was non-numeric, multi-digit, or not sufficiently digit-specific for reliable mutually exclusive clinical subdivision. The raw salvage-failure fallback subgroup could not be reliably subdivided further into complete necrosis, documented non-salvage, or other clinical categories from the available retrospective source records.

**Table 6:** Salvage success among compromise digits by key factors and complete-case denominator.

Variable	Failure	Success	*P* value
Compromise type, *n* (%)			0.241
Venous congestion (complete-case *N* = 128)	30 (63.8)	62 (76.5)	
Arterial insufficiency (complete-case *N* = 128)	12 (25.5)	15 (18.5)	
Mixed compromise (complete-case *N* = 128)	5 (10.6)	4 (4.9)	
Operation-start-referenced interval to first documentation of compromise by salvage status (h), median [IQR] (complete-case *N* = 128)	20.25 [11.98, 36.58]	14.33 [7.33, 33.70]	0.187
Any thrombolysis, *n* (%)			0.011
No (complete-case *N* = 84)	12 (36.4%)	33 (64.7%)	
Yes (complete-case *N* = 84)	21 (63.6%)	18 (35.3%)	
Bleeding/leech, *n* (%)			>0.999
No (complete-case *N* = 86)	31 (93.9%)	49 (92.5%)	
Yes (complete-case *N* = 86)	2 (6.1%)	4 (7.5%)	
Re-exploration, *n* (%)			0.558
No (complete-case *N* = 122)	45 (95.7%)	74 (98.7%)	
Yes (complete-case *N* = 122)	2 (4.3%)	1 (1.3%)	

Complete-case denominators are variable-specific. Any thrombolysis refers to thrombolysis recorded at any time during post-compromise treatment and differs from thrombolysis as an initial management component in Table 4. Missing counts were 44 for any thrombolysis, 42 for bleeding/leech, and 6 for re-exploration.

In Table 7, Model A showed no statistically significant association with the operation-start-referenced interval to first documentation of compromise (OR per hour, 0.997; 95% CI, 0.980–1.014; *p* = 0.704) or venous/mixed compromise vs. arterial insufficiency (OR, 1.500; 95% CI, 0.633–3.559; *p* = 0.357). In Model B (complete-case *N* = 84), any recorded thrombolysis was associated with lower odds of salvage success (OR, 0.243; 95% CI, 0.089–0.662; *p* = 0.006). In the sensitivity model using thrombolysis as an initial management component, thrombolysis was not significantly associated with salvage success (OR, 1.329; 95% CI, 0.385–4.589; *p* = 0.652). These complete-case exploratory associations were interpreted as non-causal because treatment decisions were clinically determined.

**Table 7:** Exploratory logistic models for salvage success after postoperative vascular compromise.

Model	Variable	OR	95% CI	*P* value
Model A: type + operation-start interval (*N* = 106 patients/128 digits; success = 81 digits; failure = 47 digits)	Operation-start-referenced interval to first documentation of compromise, per hour	0.997	0.980–1.014	0.704
Model A: type + operation-start interval (*N* = 106 patients/128 digits; success = 81 digits; failure = 47 digits)	Venous/mixed compromise vs. arterial insufficiency	1.500	0.633–3.559	0.357
Model B: operation-start interval + any thrombolysis (*N* = 68 patients/84 digits; success = 51 digits; failure = 33 digits)	Operation-start-referenced interval to first documentation of compromise, per hour	0.985	0.964–1.006	0.151
Model B: operation-start interval + any thrombolysis (*N* = 68 patients/84 digits; success = 51 digits; failure = 33 digits)	Any thrombolysis recorded	0.243	0.089–0.662	0.006
Sensitivity B2: operation-start interval + initial thrombolysis (*N* = 106 patients/128 digits; success = 81 digits; failure = 47 digits)	Operation-start-referenced interval to first documentation of compromise, per hour	0.997	0.980–1.014	0.696
Sensitivity B2: operation-start interval + initial thrombolysis (*N* = 106 patients/128 digits; success = 81 digits; failure = 47 digits)	Thrombolysis as initial management component	1.329	0.385–4.589	0.652

All salvage models are exploratory complete-case logistic models and should not be interpreted as evidence of treatment effectiveness or harm. Model B used 84 complete-case records because thrombolysis records were missing for 44 compromise digits. The per-hour estimates refer to the operation-start-referenced interval and should not be interpreted as effects per postoperative hour or as effects of delayed biological onset.

## 4 Discussion

This retrospective digit-level cohort found that postoperative vascular compromise occurred in 19.1% of replanted or revascularized digits. Venous congestion was the most common presentation, and 112 of 128 events were first documented within 48 h after the recorded operation-start timestamp. Complete amputation, vein grafting, and longer operative duration were associated with higher odds of compromise, whereas a greater number of completed arterial anastomoses was associated with lower odds. Among digits that developed compromise, approximately two thirds achieved salvage success, defined as complete or partial survival with digit preservation and without secondary amputation. These findings support risk-stratified early surveillance while reinforcing that compromise presentation, timing, and final digit survival should be reported separately. However, the operation-start-referenced timing data should not be used to define a standardized postoperative monitoring window.

The 19.1% digit-level incidence of postoperative vascular compromise in the present cohort appears lower than that reported in a recent arterial-only fingertip avulsion series, in which arterial events occurred in 28 of 194 digits and venous events occurred in 86 of 194 digits (16). However, that cohort involved distal fingertip avulsion amputations in which venous anastomosis was not feasible, so the higher compromise burden likely reflects differences in injury pattern, amputation level, and reconstructive strategy rather than a directly comparable baseline risk.

The association between complete amputation and vascular compromise is clinically plausible. Complete amputations require re-establishment of arterial inflow and venous outflow in a fully detached segment, and may therefore reflect more extensive vascular and soft-tissue disruption than incomplete injuries. Recent work in complete multi-finger amputation similarly found that operative duration and amputation plane were associated with replantation survival, supporting the relevance of surgical complexity when interpreting early postoperative perfusion risk (17). Our analysis differs by using vascular compromise as the primary endpoint, which captures a potentially reversible deterioration before final necrosis or secondary amputation.

The vein-graft association should be interpreted as a marker of injury complexity rather than as evidence that grafting itself is harmful. Interpositional grafting may be used in more complex injury patterns, and one large cohort found that arterial graft use itself was not associated with lower digit survival (18). Vein grafts are commonly required when direct repair is not feasible because of segmental vessel loss, crush, avulsion, or poor vessel quality. A 2026 meta-analysis also found vein graft use to be associated with postoperative necrosis after digital replantation, supporting grafting as a marker of higher-risk injury and reconstruction contexts (19). In practice, the finding may be most useful for postoperative triage: grafted digits should be considered high-monitoring-priority digits.

The predominance of venous compromise is consistent with the practical challenges of establishing and maintaining venous outflow in small replanted digits. Arterial-only and distal fingertip strategies can be successful in selected settings, but venous congestion remains a major postoperative management problem, especially when venous repair is limited or impossible (16). The median operation-start-referenced interval to first documentation of compromise was approximately 16 h, and most events were first documented within 48 h after the recorded operation-start timestamp. Because this measure included a variable intraoperative period, it cannot define a standardized 48-h postoperative surveillance window; nevertheless, careful early assessment of color, temperature, turgor, capillary refill, bleeding characteristics, and pain remains clinically appropriate. However, the optimal duration and cost-effectiveness of inpatient monitoring remain debated because vascular compromise and successful salvage occur only in a subset of replanted digits (20).

Because post-compromise management was individualized rather than protocol mandated, recorded interventions reflected clinical severity, suspected mechanism, persistence of compromise, and surgeon judgment. Detailed treatment protocols and escalation thresholds were not consistently retrievable from the retrospective records. Re-exploration was recorded in only 3 of 122 digits with available data, but the records did not permit reliable categorization of why re-exploration was not performed in other cases. Therefore, the low recorded re-exploration rate should be interpreted as a description of local practice and available documentation rather than as evidence regarding eligibility for or effectiveness of surgical salvage.

The salvage analysis should be read cautiously. Thrombolysis was associated with lower odds of salvage success in the complete-case model, but this almost certainly reflects confounding by indication: clinicians are more likely to use thrombolysis for more severe, persistent, or less responsive compromise episodes. The finding should therefore not be used to infer treatment harm. A retrospective review of troubled replantations also illustrates the heterogeneous management decisions and outcomes after postoperative vascular compromise (21). Similarly, the very low rate of re-exploration in this cohort limits comparison with studies reporting active surgical salvage for vascular insufficiency, including reports in which early revision increased survival among compromised replants (22).

The inverse association between severe contamination and vascular compromise is counterintuitive and should not be interpreted as protective. In a sensitivity analysis excluding unknown contamination status, the direction was similar but should still be interpreted cautiously. Possible explanations include coding differences, selection effects, residual confounding, correlations with injury mechanism or replantation candidacy, and operative strategy decisions not captured in the model.

Recent evidence on post-replantation failure has emphasized injury severity, ischemia, thrombosis, and grafting-related complexity. In the 2026 systematic review and meta-analysis, smoking, ischemia time of at least 8 h, crush injury, thrombosis, and vein grafting were associated with postoperative necrosis after digital replantation (19). A 2025 complete multi-finger amputation cohort reported that operative duration and amputation plane were associated with replantation survival (17). Compared with those survival or necrosis endpoints, the present study used early postoperative vascular compromise as the primary endpoint, which may explain why vein grafting and operative duration were prominent while smoking and ischemia time were not retained as major independent signals.

The study has several strengths. It separated patient-level baseline description from digit-level surgical exposure analysis, used patient-cluster robust standard errors, and reported compromise presentation, operation-start-referenced timing, management, and salvage denominators transparently. It also used a predefined salvage-outcome rule and distinguished thrombolysis as an initial management component from any recorded thrombolysis during post-compromise treatment. These features provide information beyond a survival-only analysis, although interpretation of the timing data remains constrained by the available operation-start time origin.

This study has several limitations. First, the single-center and retrospective observational design precludes causal inference and limits external generalizability. All clinical treatments were determined by individualized clinical judgment rather than randomized allocation; therefore, treatment-outcome associations, especially thrombolysis, are susceptible to confounding by indication and residual confounding. Vein grafting and longer operative duration may reflect injury severity, segmental vessel loss, reconstructive complexity, surgeon fatigue, or other unmeasured factors rather than independent causal effects. An additional limitation concerns the timing measure. The operation-start timestamp was the only consistently available time origin, whereas uniform timestamps for reperfusion, completion of the final vascular anastomosis, completion of surgery, or initiation of postoperative monitoring were unavailable. Consequently, the operation-start-referenced interval included a variable intraoperative period and represented the time of first clinical documentation rather than the biological onset of vascular compromise. It may also have been influenced by surveillance frequency and documentation delay. These data therefore cannot define a standardized postoperative monitoring window or establish that compromise biologically developed within the first 48 postoperative hours. Second, operative duration and ischemia time were recorded at the operation or patient level and may partly reflect the number of injured digits and overall case complexity in multi-digit injuries. Although a single-digit sensitivity analysis showed a positive direction for operative duration, the association was no longer statistically significant; therefore, operative duration should be interpreted primarily as a marker of surgical complexity rather than as an independent digit-specific causal factor. Third, the study combined complete replantation with incomplete amputation or devascularized digits requiring revascularization; although stratified descriptive results were added, subgroup modeling was limited by event counts. Fourth, the multivariable model was intended for association estimation rather than clinical prediction; therefore, discrimination, calibration, decision thresholds, and bootstrap validation were not used to support a clinical risk score. Finally, this study focused on early postoperative vascular compromise; anesthetic technique, vasopressor exposure, ICU interventions, long-term functional recovery, patient-reported outcomes, and rehabilitation outcomes were not evaluated. Large multicenter prospective cohort studies with standardized monitoring and salvage protocols are warranted.

In addition, detailed post-compromise treatment protocols, drug dosing, treatment duration, escalation thresholds, and reasons for not performing re-exploration were not consistently retrievable, which further limits interpretation of treatment-outcome comparisons.

## 5 Conclusions

In this single-center retrospective cohort of 671 replanted or revascularized digits, postoperative vascular compromise occurred in 128 digits (19.1%). Venous congestion was the most common presentation, and most events were first documented relatively early in the operation-start-referenced interval. However, this timing measure included variable intraoperative time and did not define a standardized postoperative monitoring window. Complete amputation, vein grafting, longer operative duration, and fewer completed arterial anastomoses were associated with vascular compromise, while salvage success was achieved in 63.3% of compromise digits. These findings support careful early surveillance for higher-risk digits and provide a practical framework for future prospective studies of vascular-compromise prevention and salvage; treatment-outcome associations should be interpreted as exploratory rather than causal.

## Data Availability

The datasets presented in this article are not readily available because the datasets generated and/or analyzed during the current study are not publicly available due to patient privacy and institutional restrictions, but are available from the corresponding author on reasonable request and with permission from Ningbo No. 6 Hospital. Requests to access the datasets should be directed to Jie Zhang, nbzj66@sina.com.

## References

[1] Pet MA Ko JH. Indications for replantation and revascularization in the hand. Hand Clin. (2019) 35(2):119–30. 10.1016/j.hcl.2018.12.003 30928045

[2] Cho HE Kotsis SV Chung KC. Outcomes following replantation/revascularization in the hand. Hand Clin. (2019) 35(2):207–19. 10.1016/j.hcl.2018.12.008 30928052 PMC6446589

[3] Chang MK Lim JX Sebastin SJ. Current trends in digital replantation—a narrative review. Ann Transl Med. (2024) 12(4):66. 10.21037/atm-23-1515 39118941 PMC11304438

[4] Dec W. A meta-analysis of success rates for digit replantation. Tech Hand Up Extrem Surg. (2006) 10(3):124–9. 10.1097/01.bth.0000225005.64605.17 16974215

[5] Sebastin SJ Chung KC. A systematic review of the outcomes of replantation of distal digital amputation. Plast Reconstr Surg. (2011) 128(3):723–37. 10.1097/PRS.0b013e318221dc83 21572379 PMC3163033

[6] Shaterian A Rajaii R Kanack M Evans GRD Leis A. Predictors of digit survival following replantation: quantitative review and meta-analysis. J Hand Microsurg. (2018) 10(2):66–73. 10.1055/s-0038-1626689 30154618 PMC6103763

[7] Breahna A Siddiqui A Fitzgerald O Connor E Iwuagwu FC. Replantation of digits: a review of predictive factors for survival. J Hand Surg Eur. (2016) 41(7):753–7. 10.1177/1753193415624663 26763268

[8] Zhang Z Credico P Bristol S Macadam S. Determinants of success in single- and multi-digit replant. Plast Surg (Oakv). (2023) 31(1):53–60. 10.1177/22925503211024767 36755824 PMC9900039

[9] Hamada D Suzuki H Muramatsu K Zenke Y Kawasaki M Fushimi K. Analyzing attempt and success factors for amputated digit replantation in Japan using the diagnosis procedure combination database. Sci Rep. (2024) 14:12156. 10.1038/s41598-024-62879-2 38802545 PMC11130330

[10] Lee BI Chung HY Kim WK Kim SW Dhong ES. The effects of the number and ratio of repaired arteries and veins on the survival rate in digital replantation. Ann Plast Surg. (2000) 44(3):288–94. 10.1097/00000637-200044030-00007 10735221

[11] Prsic A Friedrich JB. Postoperative management and rehabilitation of the replanted or revascularized digit. Hand Clin. (2019) 35(2):221–9. 10.1016/j.hcl.2019.01.003 30928053

[12] Chen C Chen J Liu WC Tuaño KR. Overview and management of complications after digital replantations. J Hand Surg Eur. (2024) 49(2):167–76. 10.1177/17531934231212394 38315131

[13] Reissis D Geoghegan L Sarsam R Sing QY Nikkhah D. Perioperative thromboprophylaxis in digital replantation: a systematic review. Plast Reconstr Surg Glob Open. (2020) 8(5):e2806. 10.1097/GOX.0000000000002806 33154865 PMC7605889

[14] Chen C Scott F Ipaktchi KR Lauder A. Postoperative digit and hand replantation protocols: a review of the literature. J Am Acad Orthop Surg. (2021) 29(15):e732–42. 10.5435/JAAOS-D-20-01176 34185029

[15] Rönkkö H Neergård Sletten I Liv Hansen K Ryhänen J Pietreanu M Jokihaara J. Indications, anaesthesia and postoperative protocol for replantation and revascularization in the hand in nordic countries. J Hand Surg (Eur Vol). (2023) 48(1):46–51. 10.1177/17531934221123427 36165410 PMC9773149

[16] Weng Z Ainiwaer A Ding W. Arterial-only anastomosis for fingertip avulsion amputations: survival rates and functional outcomes in a retrospective study of 194 digits. BMC Surg. (2026) 26:309. 10.1186/s12893-026-03692-8 41888796 PMC13141435

[17] Pang X Xu P Huang Y Liu C Shen Y Zhang Z. Analysis of risk factors for post-replantation necrosis in complete multi-finger amputation. JPRAS Open. (2025) 46:637–45. 10.1016/j.jpra.2025.10.006 41280471 PMC12636383

[18] Lee ZH Klifto CS Milone MT Cohen JM Daar DA Anzai L. Survival after digit replantation and revascularization is not affected by the use of interpositional grafts during arterial repair. Plast Reconstr Surg. (2019) 143(3):551e–57e. 10.1097/PRS.0000000000005343 30601326

[19] Yu F Lu L Zhou Y Wang R Zheng H. Risk factors associated with postoperative necrosis after digital replantation: a systematic review and meta-analysis. Front Surg. (2026) 13:1795247. 10.3389/fsurg.2026.1795247 42052376 PMC13111292

[20] Elmaraghi S Israel JS Gander B. Systematic review of replant salvage and cost utility analysis of inpatient monitoring after digit replantation. J Hand Surg Am. (2022) 47(1):32–42.e1. 10.1016/j.jhsa.2021.07.024 34548183

[21] Chia DSY Tay SC. A retrospective review of troubled replantations. Hand Surg. (2015) 20(1):127–32. 10.1142/S0218810415500185 25609286

[22] Gunturk OB Kayalar M Bali U Ozaksar K Toros T Gurbuz Y. Clinical outcomes of salvage revision surgery following finger replantation with vascular insufficiency: a retrospective study. Acta Orthop Traumatol Turc. (2020) 54(6):577–82. 10.5152/j.aott.2020.19016 33423987 PMC7815223

